# Fifty-Ninth Annual Meeting of the New York State Medical Society

**Published:** 1866-02

**Authors:** 


### Miscellaneous.

### Fifty-Ninth Annual Meeting of the New York State Medical Society.

The Fifty-Ninth Annual Session of the New York State Medical Society commenced at the City Hall, Albany, Tuesday morning, 6th instant, at 11 o'clock.

The Society was called to order by Dr. Henry W. Dean, of Rochester, President, and prayer was offered by Rev. Mr. Bidwell.

The President then delivered his inaugural address.

Dr. Cobb moved that a committee of three be appointed to extend an invitation to such members of the Legislature as belong to the medical profession, to attend the meetings of the Society during its sessions. Adopted, and Drs. Cobb, Williams and Bissell were appinted.

The Chair announced the Standing Committees.

There were present seventy-five permanent members, sixty-five delegates from County Societies and Institutions, and about fifty invited members.

Dr. Kennedy, of New York, offered the following:

*Resolved*, That the Medical Society of the State of New York, now in Convention, in view of the importance of the bill now before the Legislature, and which has passed the Senate, known as the "New York Health Bill," very respectfully ask the Assembly to concur with the Senate, in order that the said bill may become a law.

*Resolved*, That a copy of the above resolution be sent to the Speaker of the Assembly.

Dr. Kennedy said that he thought the adoption of this resolution by this body would have the desired effect. Such a bill was needed very much in anticipation of the cholera.

Dr. B. P. Staats would like to have the bill read before the Society that the members might know what was proposed by it. He had heard that the bill had been "doctored" in the Senate. He, therefore, preferred having the matter referred to a committee.

Dr. J. T. Williams moved that this subject be referred to a committee of five to be appointed by the President, and that they report to-morrow. Adopted, and Drs. Willard Parker, Stephen Smith, Kennedy, Agnew and J. T. Williams were appointed such committee.

Dr. Lawrence McKay, of Rochester, read a paper entitled "The Gingeral Margin as a Diagnostic Sign."

On motion of Dr. Corliss, Dr. Willard Parker made a few appropriate remarks regarding the Health Bill, now before the Legislature.

Drs. Kennedy, B. P. Staats and J. T. Williams participated in the debate.

Dr. Squibbs, of Brooklyn, read a very interesting paper entitled "An Appeal for the Materia Medica," accompanied by the following:

He said: In order that there may be some organization in this Society that may, at least, serve to keep this subject in useful remembrance, the following resolutions are offered for consideration:

*Resolved*, That a committee of four, to be called "The Committee on Pharmacology," be appointed by the President, to hold office until the annual meeting of 1871.

*Resolved*, That it shall be the general duty of the members of this committee individually to accumulate knowledge upon medicinal agents and their application, and to report the result of their researches separately, through the chairman of the committee, annually to this society.

*Resolved*, That it be a special duty of this committee to take charge of the interests of this Society in the United States Pharmacopœia, and to collect, arrange, preserve and transmit all accessible information and knowledge that may be useful in the next decennial revision of that work in 1870, and to carry out the general provisions and requests of the National Convention of 1860, as they apply to this Society as a constituent of the National Convention of 1870.

*Resolved*, That this committee report to the Society, at its annual meeting in 1870, the names of three members of the committee, who, if confirmed by the action of the Society, shall serve as the representative delegates of the Medical Society of the State of New York in the National Convention of 1870, for revising the United States Pharmacopœia, to be held in Washington, on the first Wednesday of May, 1870; and that the delegation thus constituted be authorized and directed, on behalf of this Society, to conform to the rules adopted by the last National Convention, to facilitate the organization and effect the objects of the next one.

*Resolved*, That this committee shall apply to the Society to supply any vacancies that may occur in its members.

Dr. Wm. B. Brobins moved that the paper be received by the Society. Adopted.

Dr. Sayre moved the adoption of the resolutions accompanying the paper of Dr. Squibbs. Adopted.

The President appointed the following gentlemen the Pharmacological Committee: Dr. Ed. R. Squibbs, Howard Townsend, C. Green, Manlius Smith, Fowler.

Dr. Sayre moved that the paper and resolutions presented by Dr. Squibbs be presented to the different State Medical Societies, with the request that they take similar action. Adopted.

The Treasurer, Dr. Quackenbush, presented his annual report, which was accepted, and, on motion of Dr. Corliss, referred to the usual committee for examination.

Memorials were presented from the New York and Erie County Medical Societies.

On motion of Dr. Vanderpool, the memorials were endorsed by the Society and referred to a committee of three, to confer with the Legislature.

Dr. Gurdon Buck, of New York, read a paper illustrative of cases, one of "Destruction of the body of the lower jaw, with extensive disfigurement of the face, from a shell wound;" also, two other cases of deformity.

Dr. E. R. Peaslee, of New York, read a paper upon "Retroflexion of the unimpregnated Uterus."

The following papers were recommended by the Business Committee to be read by title and referred to the Committee of Publication:

"An adaptability of the Hospital and Cottage plan to the treatment and management of the insane poor, as illustrated by the colony of Fitz James, at Clermont in France, by Charles A. Lee, M. D."

Also, "An enquiry into the mode of propagation of cholera, with facts and reasons in favor of the theory of its transmission by choleraic stools, and in no other manner, with suggestions in regard to the proper preventive measures to be used in case the disease should again appear among us, by Charles A. Lee, M. D."

Also, "A case of Acute Enterites, treated by B. G. McCabe, M. D., Monticello, Sullivan county."

The Committee appointed on the Inaugural Address of the President reported as follows:

That the first matter claiming the attention of the committee is the one so feelingly alluded to by the President to the untimely death of our late Secretary, Dr. Sylvester D. Willard. With but limited knowledge of Dr. Willard, except in his official capacity, it is obvious that your committee cannot speak appropriately of his life and character; they therefore suggest that the President designate some one of the many personal friends of our late lamented Secretary to prepare a suitable memorial of him and transmit it to the Committee of Publication in time to have it appear in the next volume of our transactions.

Our President notices very justly the great want of interest taken by a too large portion of the profession in medical matters, as manifested by neglecting to identify themselves with, and in many instances ignoring the County Societies, and expresses the hope that some measures may at once be inaugurated whereby there may be brought to the aid of the Society the intent, experience and influence of a large class of medical men not belonging to any medical society or connected with various organizations not having by law any relations with this Society. Your committee cannot see that any legal remedy can or should be brought to bear on the existing state of things; nor do they know that any was contemplated by our President. We can only appeal to the stirling good sense of those gentlemen, and urge upon them as a duty, the uniting themselves with the Societies entitled to representation in this body, and thereby building up, or greatly strengthening, the local organizations, and enabling them to send us more representative men who will co-operate with us in the work of elevating the position, and enlarging the power for good, for the entire medical profession.

As to the suggested change in the manner of electing permanent members according to a pro rata of the medical population, rather than by the mode now in operation, which is based on political divisions of territory, and which gives us two members annually from each Senatorial District, your committee think that the mode suggested would be eminently just, and would eventually result in our securing a larger number of active, intelligent, working members than we are likely to have by the operation of the present mode.

All statutory provisions for the protection of the profession having been removed, it seems altogether reasonable that the power should be given to the local medical societies to determine the conditions of membership, and to discipline their members for any violation of that code of ethics under which we all act, with an appeal to the State Medical Society, as the "Tribunal to which all differences originating in the County Societies shall be referred for ultimate and final adjustment."

Your Committee express the hope that measures may be taken whereby there shall be secured the enactment of such laws as the existing state of things in our profession seem to demand.

All of which is respectfully submitted.

EDWARD L. BEADLE, } Committee.

JAMES KENNEDY, }

Dr. Williams, from Committee on Health Bill, reported by resolution, as follows:

*Resolved*, That the State Medical Society now in session, does hereby earnestly urge the Assembly to pass, at the earliest day, a Health Bill which shall retain the general sanitary provisions and regulations contained in the bill which recently passed the Senate; as to the mode or manner of appointing the Commissioners to execute said law, the Society offers no suggestions, leaving this wholly to the wisdom of the Legislature.

*Resolved*, That a copy of the above resolution be sent to the Speaker of the Assembly at the earliest practicable moment. — Adopted.

Dr. Crandall offered the following, which was adopted:

*Resolved*, That in accordance with the suggestions of the Committee on the Inaugural Address, the President is hereby authorized to appoint one or more members of this Society, residing in Albany, to prepare a suitable memorial of the late Secretary, Sylvester D. Willard, and that the same be published in the forthcoming transactions.

Dr. J. H. Curry offered the following:

*Whereas*, This Society wishes to express farther its appreciation of the life and character of S. D. Willard, M. D., late Secretary of this Society, and to show in some measure their sympathy with his numerous friends not connected with the profession; therefore,

*Resolved*, That the members of this Society do request the biographer of the late Dr. Willard to issue five hundred extra copies of said biography, together wilh the steel plate of his likeness, and that the members of this Society will pay each the sum of $—— to defray the expenses of the same.

The Chair appointed Drs. Townsend, Hun and Bailey a committee to collect subscriptions for the above object.

Dr. Brinsmade, from the committee appointed to draft appropriate resolutions in reference to the decease of Dr. Blatchford, of Troy, reported as follows:

*Resolved*, That this Society has heard, with profound sorrow, the announcement of the death of our late distinguished brother member and former president, Dr. Thomas W. Blatchford, of Troy; that his eminent abilities, high professional attainments, and social virtues, had won for him the esteem and regard of the medical profession of the State, and secured the respect and confidence of the community in which he was best known; and that, in his decease, this Society is called upon to mourn the loss of one of its most honorable and useful members, and the city in which he lived, and labored, add died, one of its best men.

*Resolved*, That this Society extend an expression of its condolence to the widow and children of the deceased, and that a copy of these resolutions be sent to them by the Secretary of this Society.

*Resolved*, That Dr. Stephen Wickes, of Orange, N. J., be requested to write a biography of the late Dr. Thomas W. Blatchford, and present it for publication in the next volume of the Transactions of this Society.

Adopted.

Then, on motion, the Society adjourned to meet at eight o'clock, in the Assembly Chamber, to hear the annual address of the President.

Agreeable to adjournment, the Society met in the Assembly Chamber at eight in the evening. The meeting was called to order by Dr. Hutchison, Vice President, after which he introduced Dr. Dean, who delivered a very able and finished address.

When the Society adjourned to meet at their usual place of assembling at nine o'clock Thursday morning.

Dr. Hutchison moved that the committee appointed to draft suitable resolutions expressing the sense of the Society relative to the death of Dr. Mott, be authorized to prepare such resolutions as they may think proper, and send them to the Committee on Publication, to be published in the Transactions of this Society. Adopted.

Dr. Shipman, from the committee appointed to draft suitable resolutions on the death of Dr. Wm. Taylor, of Manlius, Onondaga county, reported the following:

*Whereas*, We have heard with profound regret the death of Dr. Wm. Taylor, of Manlius, Onondaga county, one of the permanent members of this Society and formerly its President, therefore

*Resolved*, That the loss of such a man is one that all classes of community most deeply deplore, as he was one who had spent a long life in the service of his profession, faithfully and conscientiously laying aside all selfish feelings to do his duties to the sick and suffering.

*Resolved*, That as a man, Dr. Taylor was a credit and an honor to the human race; in him was combined in harmonious proportions, all the virtues that adorn a man and stamps the Christian gentleman.

*Resolved*, That while we cherish his memory we will endeavor to imitate his virtues as the best means of keeping alive his enduring remembrance. Adopted.

Dr. Bissell offered the following, which was adopted:

*Whereas*, The threatened approach of epidemic cholera is creating alarm and fear among all classes of people throughout the State; and,

*Whereas*, In the opinion of this Society cholera may be mainly if not entirely prevented from becoming *epidemic* in any city, town or locality, by the adoption and rigid enforcement of proper hygenic measures; and,

*Whereas*, It is due from this Society to take such action to protect our people throughout the State as a duty to our profession, and the safety and welfare of the citizens demand; therefore,

*Resolved*, That this Society concur in the views taken and the recommendations made by the "Council of Hygiene and Public Health of the citizens of New York, to protect the people of that city against the introduction and spreading of Asiatic cholera; the causes which will produce epidemic cholera, and the hygienic measures which will prevent its deadly march in that city will produce like effects in every other part of the State.

*Resolved*, That the Council of Hygiene and Public Health of said city are entitled to the thanks of the medical profession and people of the State, for their full and able report on Epidemic Cholera, adopted Nov. 14th, 1865, and that a copy of the same should at once be placed in the hands of all Common Councils, town authorities, and Boards of Health within the State limits, for their direction and guidance in the case of preventive measures, before the cholera shall visit their localities.

Dr. A. N. Bell moved that the same acknowledgments be tendered to Drs. Sayre, Murphy and Swinburne, and to the Mayor of New York, for their efforts to exclude and prevent cholera, as published in a pamphlet for public distribution, under date of November, 1865, and that this pamphlet be distributed in the same manner as that of the report of the Citizens' Association. Adopted.

Dr. Wheeler, of Massachusetts, made some very interesting remarks expressive of his appreciation of the Society. He also illustrated a case of "Urinary Calculi."

Dr. Parker, from the Committee on Prize Essays, reported as follows:

*To the Medical Society of the State of New York:*

The Committee on the Merritt Cash Prize appointed by your honorable body being also the Committee on the Brinsmade Prize, beg leave most respectfully to report as follows; and first with relation to the

MERRITT CASH PRIZE.—Your committee are pained to be compelled to announce that no competitative essays for the above prize have been received during the past year. Notwithstanding the fact that the subject selected has been one of such dominant importance in the medical history of our great civil war, and the opportunities for studying it, whether in the field and among large bodies of troops, or upon individuals in the multitude of hospitals scattered throughout the country, have made this subject familiar to every surgeon in the public service, the prize—now doubled in amount by accumulation of two years, still remains uncompeted for.

Considering the very extensive field of operations upon which our armies were employed, extending from Pennsylvania to Texas, and from the Atlantic ocean to the western tributaries of the Mississippi, thus embracing an area of over 400,000 square miles, and presenting every variety of climate, whether marine, mountain or inland, together with the modifying influence of soil, vegetation and water courses upon the development, progress and termination of diseases, your committee, in selecting the subject of chronic diarrhœa for this prize, believed that they had chosen a rich field for competition, and confidently hoped, therefore, to elicit from the medical profession a large number of essays, from among which to select a successful candidate. They had greatly desired that some contribution to the medical history of a disease, which was the special scourge of our armies, might, for the honor of this Society, appear among its printed Transactions; and for this end retained the subject a second year as a further invitation to competitors. In this hope your committee have been sadly disappointed, and they feel compelled, therefore, to withdraw the subject, unless your honorable body should otherwise order.

Brinsmade Prize.—For this prize but one essay has been presented, and inasmuch as this does not comply with the rules specially laid down by the founder of the prize, nor fill the scope of requirements exacted by him, your Committee do not feel themselves authorized to receive the essay, and cannot, consequently, express any opinion upon its merits.

Your Committee cannot close their report without paying a passing tribute to the memory of their lamented colleague, the late Dr. Thomas W. Blatchford, known to this Society and to the profession throughout the country, as one of its most distinguished members and ornaments, his long life and usefulness has left behind it a memory without soil and without tarnish. To a mind of massive proportions, he united the rare graces of a refined scholarship, and brought to the elucidation of a scientific problem a logical power which enabled him to grapple and master their elements as by intuition. The candor and inflexible honesty of his character compelled the admiration and won the esteem of all. He was a true man, and the well-rounded outlines of a symmetrical nature closing in a wealth of personal merit, which affected no ostentation of display. As one of the pillars of this Society, his loss will not soon be repaired, while to those who enjoyed the privilege of his friendship, his memory will be endeared through life. *Extinctus amabitur idem.* The report was accepted.

Dr. Vanderpool moved that the same Committee be continued, and that the subject on essays be referred back to that Committee to act according to their judgment. Carried.

Dr. Squibbs, from the Nominating Committee, presented the following, which was adopted unanimausly:

The Committee on Nominations beg leave to recommend the following names for election to fill the offices and delegations of the ensuing year:

*For President*—Joseph C. Hutchins, M. D., of Brooklyn.
*For Vice President*—Julien T. Williams, M. D., of Dunkirk.
*For Secretary*—William H. Bailey, M. D., of Albany.
*For Treasurer*—J. V. P. Quackenbush, M. D., of Albany.

*Delegates to the American Medical Association.*—Drs. T. C. Brinsmade, D. P. Bissell, H. W. Dean, C. C. Wyckoff, A. L. Saunders

Samuel G. Wolcott, J. C. Hutchison, James L. Banks, Edward Hall, L. J. Tefft, James Ferguson, Seth Shove, H. H. Langworthy, C. Green, J. K. Chamberlayne, F. Jacobs, E. H. Parker, G. J. Fisher, H. A. Carrington, Harvey Jewett, S. Oakley Vanderpool, John R. Van Kleck, Wm. H. Bailey, Thomas Hun, C. M. Crandall.*

* We are obliged to greatly abridge the report of the Society for want of space in our pages.

